# The Role of Fractional Excretion of Uric Acid in the Differential Diagnosis of Hypotonic Hyponatraemia in Patients with Diuretic Therapy

**DOI:** 10.7759/cureus.7762

**Published:** 2020-04-21

**Authors:** Vincenzo Bassi, Olimpia Fattoruso

**Affiliations:** 1 Internal Medicine, San Giovanni Bosco Hospital, ASL Napoli 1 Centro, Naples, ITA; 2 Pathology, San Giovanni Bosco Hospital, ASL Napoli 1 Centro, Naples, ITA

**Keywords:** hyponatraemia, diuretics, thiazide diuretics, furosemide, potassium canrenoate, syndrome of inappropriate antidiuresis, syndrome of inappropriate antidiuretic hormone secretion

## Abstract

Hyponatraemia is the most common electrolyte imbalance found in hospital population and worldwide thiazide and loop-diuretics are among the most widely used drugs. Syndrome of inappropriate antidiuresis diagnosis (SIAD) is complicated in the presence of diuretic therapy due to the misleading clinical assessment of the extracellular volume status, and in order to make SIAD diagnosis it is often necessary to withdraw diuretic therapy. Our study aimed to investigate the diagnostic role of these alternative markers of volume status, serum uric acid (sUA) and fractional excretion of uric acid (FEUA), in hyponatraemic patients treated with different diuretic drugs. Eighty-nine patients were enrolled with the diagnosis of SIAD, diuretic-induced hyponatremia (DIH, treated with furosemide and potassium canrenoate) or thiazide-induced hyponatremia (TIH, treated with hydrochlorothiazide, metolazone or indapamide) and investigated with receiver operating characteristic analysis and a sensitivity test. Our results show that FEUA discriminated better than sUA between SIAD and DIH patients (area under curve 0.96, <0.001 vs. 0.88, <0.001) while it was a poor marker to discriminate between SIAD and TIH (0.65, NS vs. 0.67, NS). In conclusions, FEUA is an excellent marker to discriminate SIAD vs. sodium depleted patients treated with furosemide and/or potassium canrenoate while the diuretic withdrawal, beyond obtaining a serum Na normalization, is still mandatory for differential diagnosis of sodium depleted patients affected by thiazide-induced hyponatraemia.

## Introduction

Hyponatraemia is a frequent electrolyte imbalance occurring in up to 25-30% of hospitalized patients where syndrome of inappropriate antidiuresis (SIAD) is the most common etiology present in nearly 35% of hyponatremic inpatients [1, 2]. The actual guidelines suggest different algorithms to help the physicians in the differential diagnosis of hypotonic hyponatraemia, but a potential bias is coming from the clinical assessment of the volemic status, especially in diuretic-treated patients [3-5].

Worldwide thiazide and loop-diuretics are among the most widely used drugs blocking the respective ion-cotransporters at different sites with inhibition of tubular Na reabsorption and increase of urine Na and water excretion. The SIAD diagnosis is complicated in the presence of diuretic therapy due to the misleading clinical assessment of the extracellular volume status [6]. In many clinical trials, the presence of diuretics arbitrarily excluded the diagnosis of SIAD in hyponatremic patients [7, 8]. In order to make SIAD diagnosis it is often necessary to withdraw diuretic therapy up to 10 days [9].

Fenske et al. demonstrated that the fractional excretion of uric acid (FEUA) could discriminate between SIAD and diuretic-induced hyponatraemia in these confounding patients but only 13% of enrolled patients were treated with thiazide therapy [5].

The aim of our study was to investigate the diagnostic role of these alternative markers of volume status, serum uric acid (sUA) and FEUA, in hyponatremic patients treated with different diuretic drugs.

## Materials and methods

Retrospectively, between March 2016 and October 2018, 122 patients, older than 18 years, presenting a serum sodium concentration <130 mEq/L, serum osmolality <280 mEq/Kg, were consecutively identified and 89 patients enrolled with the diagnosis of SIAD or diuretic-induced hyponatraemia. Serum hyponatraemia was confirmed using the hemogasanalysis study (direct measurement) to exclude bias of dilution.

Further eligibility criteria were a normal kidney, thyroid and adrenal function, urine osmolality >100 mEq/kg, a sufficient dietary daily intake (at least 10 mEq/kg as solutes), no polydipsia story and, if treated, a diuretic therapy (hydrochlorothiazide, metolazone, indapamide, furosemide and potassium canrenoate) longer than three months. The presence of liver cirrhosis and acute neurological diseases (potentially complicated by cerebral salt wasting syndrome] were non-eligibility criteria in consideration of the interference with FEUA values [10, 11].

Particular attention was paid to the medical anamnesis and the pharmacotherapy of the enrolled patients. The effective arterial blood volume (EABV) status was investigated using the parameters of change of pulse rate and blood pressure on supine and upright body position in enrolled patients [12]. Hypervolemic patients, usually with a congestive heart failure diagnosis, were identified with a clinical examination of edema presence. The normalization of serum Na after the withdrawal of diuretic therapy determined the diagnosis of diuretic-induced hyponatraemia. In doubtful cases a fluid challenge test (2 L isotonic saline in 24 h) was performed and a serum sodium increase >5 mEq/L with a ∆FENa <0.5% identified a mild hypovolemic status of a non-SIAD patient.

Laboratory parameters were tested with automated clinical analysis, using ion-selective electrodes (indirect measurement, COBAS 6000 Analyzer Series, Roche, Switzerland). A blood sample was taken in the morning contemporary to a urine spot sample to test Na, K, uric acid, glucose, blood urea nitrogen (BUN), creatinine and then calculate the different fractional excretions (FENa, FEUA).

We identified a SIAD and a non-SIAD diuretic-induced hyponatraemia group.

SIAD diagnosis was based on the classical Schwartz and Bartter criteria such as a serum hyponatraemia <130 mEq/L, a serum osmolality <280 mEq/kg (either measured or calculated with the formula: Posm = Na [mEq/L] x 1.86 + glucose/18 [mEq/L] + BUN/6 [mEq/L] + 9), clinical euvolemia, urine sodium concentration >30 mEq/L, urine osmolality >100 mEq/kg (either measured or calculated with the formula: Posm = Na [mEq/L] x 1.86 + glucose/18 [mEq/L] + BUN/6 [mEq/L] + 9) [13].

In the presence of coexistent diuretic therapy the persistence of hyponatraemia <130 mEq/L to the diuretic withdrawal confirmed the diagnosis of SIAD. Instead, the non-SIAD diuretic-induced hyponatraemia diagnosis was based on hypovolemic or hypervolemic clinical status and normalization of hyponatraemia after diuretic withdrawal.

The non-SIAD diuretic-induced group was divided into two subgroups:

Diuretic-induced hyponatraemia (DIH, patients treated with furosemide and/or potassium canrenoate) and thiazide-induced hyponatraemia group (TIH, patients treated with hydrochlorothiazide, metolazone or indapamide).

All data analysed retrospectively were collected as part of routine diagnosis. Written informed consent was obtained from all the patients before participation.

Statistical analysis

The means of different groups were tested with a nonparametric Kruskal-Wallis test. Group comparisons between patients with and without SIAD were performed with the Student’s test after testing for equality of variances with Levene’s test.

The accuracy of SIADH diagnosis vs. diuretic-induced hyponatraemia group was tested using receiver operating characteristic (ROC) analysis calculating area under the curve (AUC) by the nonparametric trapezoidal rule with 95% confidence interval (CI). A sensitivity analysis to identify the optimal cutoff points was performed using Wilson/Brown method. The statistical analysis was performed with Prism 8.3.0 program (GraphPad Software, San Diego, CA, USA).

## Results

The characteristics of investigated patients, different etiologies and treatments are shown in Table 1.

**Table 1:** Clinical characteristics of the investigated groups. SIAD: Syndrome of inappropriate antidiuresis; DIH: Diuretic-induced hyponatremia; TIH: Thiazide-induced hyponatremia; AVP: Vasopressin analogs. (mdd, days) indicate the mean daily dose of diuretics and mean time of withdrawal of the diuretic therapy to obtain normonatraemia. Data are mean ± standard deviation or numbers.

	SIAD Group (n = 42)		DIH Group (n = 25)	TIH Group (n = 22)	
Age	69.4 ± 11.5		72.0 ± 5.1	77.3 ± 7.2	
Sex (M/F)	23/19		12/13	10/12	
Etiology SIAD					
Neoplasia	17		-	-	
Acute infection (pneumonia)	11		-	-	
Iatrogenic (AVP analogs)	4		-	-	
Idiopathic	10		-	-	
Extracellular volume depletion	0		13	4	
Euvolemia	42		7	17	
Extracellular volume expansion		0	5		2
% of current diuretic therapy (mdd, days)		12 (28.6%)	25 (100%)		22 (100%)
Furosemide (20 mg, 3.2 d)		8	19		0
Furosemide + canrenoate (45 mg + 90 mg, 3.0 d)		4	6		0
Hydrochlorothiazide (17.1 mg, 4.3 d)		0	0		17
Indapamide (3.8 mg, 5.1 d)		0	0		3
Metolazone (4.1 mg, 3.8 d)		0	0		2

DIH and TIH patients were older compared with SIAD patients. Diuretic therapy was present in 28.6% of the SIAD patients where the dominant cause was neoplasia (17/42 patients) and infections, usually pneumonia (11/42 patients). In DIH patients vs. TIH the extracellular volume depletion was a dominant finding (13/25 vs. 4/22 patients). The fluid challenge test was performed in 29 patients (12 patients identified as SIAD, 13 patients as DIH and four patients as TIH). A significant difference was found among SIAD and diuretic-treated patients in BUN, serum UA and urine Na value while FEUA was significantly different only in SIAD vs. DIH patients (Table 2).

**Table 2:** Laboratory parameters in the investigated study groups. Data are mean (standard deviation) or numbers. * Indicates a P-value < 0.05 vs. SIAD. SIAD: Syndrome of inappropriate antidiuresis; DIH: Diuretic-induced hyponatremia; TIH: Thiazide-induced hyponatremia; FE: Fractional excretion.

	SIAD	DIH	TIH	P-value
Serum				
Na (135-145 mEq/L)	126.6 (6.9)	128.4 (2.8)	127.4 (5.8)	NS
K (3.5-4.5 mEq/L)	4.1 (0.5)	3.8 (0.8)	4.2 (1.1)	NS
Creatinine (0.5-1.2 mg/dl)	0.5 (0.2)	1.0 (0.4)	0.9 (0.3)	NS
BUN (20-50 mg/dl)	24.2 (10.4)	55.8 (21.8)*	53.1 (32.3)*	0.01
UA (3.4-7.0 mg/dl)	2.5 (1.1)	8.0 (3.6)*	4.4 (2.2)*	0.001
Osmolality (275-285 mEq/kg)	254.3 (12)	263.8 (15)	261.4 (13)	NS
Urine				
Na (100-200 mEq/L)	105.9 (78)	37.3 (29.9)*	51.7 (43.2)*	0.001
K (50-100 mEq/L)	28.0 (22.7)	33.0 (4.1)	27.8 (14.9)	NS
Osmolality (50-1200 mEq/kg)	487.3 (167)	497.6 (151.7)	476.6 (194)	NS
FE				
Na (%)	1.6 (0.4)	1.4 (0.6)	1.2/0.6)	NS
UA (5-9%)	16.1 (4.4)	5.9 (3.8)*	14.6 (6.6)	0.001

**Figure 1:**
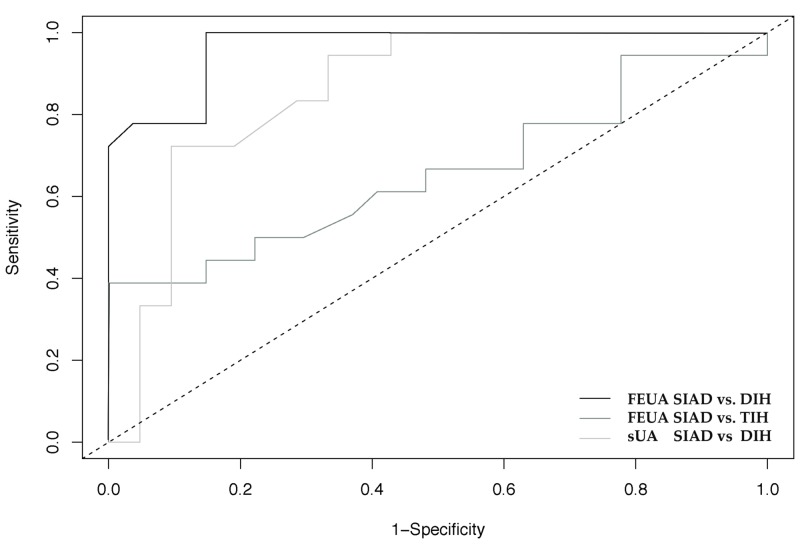
ROC analysis of the three investigated groups. Area under the curve (95% CI) FEUA SIAD vs. DIH 0.96 (0.92-1.0), FEUA SIAD vs. TIH 0.65 (0.48-0.83), sUA SIAD vs. DIH 0.88 (0.77-0.99) Comparison of the diagnostic utility (ROC analysis, area under curve: AUC) of fractional excretion of uric acid (FEUA) to differentiate among syndrome of inappropriate antidiuresis (SIAD) and diuretic-induced hyponatremia (DIH) or thiazide-induced hyponatremia (TIH) patients and serum uric acid (sUA) between SIAD and DIH. The diagonal line indicates the area of 0.5, corresponding to no informative discrimination.

The ROC analysis confirmed that FEUA discriminated better than sUA between SIAD and DIH patients (AUC 0.96, <0.001 vs. 0.88, <0.001) while it was a poor marker to discriminate between SIAD and TIH (0.65, NS vs. 0.67, NS, Figure 1).

The sensitivity analysis showed that FEUA cut-off point higher than 10% resulted in a 100% specificity and 85% sensitivity while a value less than 8% showed a 100% sensitivity and 72% specificity to identify SIAD patients (Table 3).

**Table 3:** FEUA cut off points. Different FEUA cut off points with relative sensitivity and specificity. Normal euvolemic population shows a 5-9% range. FEUA: Fractional excretion of uric acid.

FEUA	8%	9%	10%
Sensitivity	100%	85%	85%
Specificity	72%	77%	100%

## Discussion

Hyponatraemia is by far the most common electrolyte imbalance found in hospital population and a severe grade (<125 mEq/L) is detected in 2% of inpatients [2]. The natriuretic effect of diuretic therapy has a confounding role in the evaluation of EABV to obtain a differential diagnosis of hyponatraemia investigated with different algorithms. Usually, thiazide drugs increase six-fold the risk of hyponatraemia vs. non-exposed patients with an estimated incidence of 11% in the geriatric population [14]. The causes of TIH, beyond the tubular effects of thiazides on specific Na/Cl cotransporters, are water retention, both with an increase of arginine vasopressin activity and/or upregulation of aquaporin-2 expression, resembling a laboratory pattern of SIAD [15,16]. Furthermore, hypokalaemia, a frequent hallmark in thiazide-treated patients, inducing potassium depletion can increase the volume receptor release of vasopressin [17]. However, most patients in our study did not show associated hypokalaemia, probably in consideration of the low daily dose of thiazide therapy. Another possible key to explain TIH could be the genetic expression of specific prostaglandin transporters in a subset of patients prone to develop hyponatraemia [18, 19].

According to these data, the majority of patients affected by TIH are clinically euvolemic showing characteristics of extracellular volume expansion [20]. In the real world, the withdrawal of diuretics is generally considered mandatory for a correct diagnosis resulting in a longer hospitalization period and increased hospital admission costs.

Serum UA and its fraction excretion are considered useful markers of the volemic status in acute patients being on UA renal handling in the proximal tubule preserved by interferences induced by the most used diuretics [21]. Moreover, FEUA value on a urine spot sample has the advantage to avoid a 24-h urine collection resulting in a more accurate parameter [22]. Beck reported that hyponatraemia secondary to SIADH is generally associated with a serum uric acid level <4 mg/dl with an increase of FEUA [23]. These findings are dependent on a decrease in tubular reabsorption of urate whereas secretion seems to be appropriate for the low level of uricemia [24, 25].

Fenske et al. have demonstrated that FEUA cut-off value >12% (86% sensitivity and 100% specificity) is an optimal marker to discriminate between SIAD and diuretic-induced hyponatraemia. The limit of his study was the reduced number of patients treated with thiazide (only seven patients corresponding to 13% of the overall group) [5].

Our results confirm that FEUA value is a reliable marker to discriminate SIAD vs. sodium depleted DIH patients excluding the need for diuretic withdrawal and/or a fluid challenge test with a risk of water overload. The sensitivity analysis shows that FEUA cutoff point higher than 10% confirms SIAD diagnosis (100% specificity) whereas a value of less than 8% excludes it (100% sensitivity).

But instead, FEUA value is not able to identify correctly TIH vs. SIAD patients in consideration that the majority of our investigated TIH patients show effectively a euvolemic phenotype resembling SIAD pattern (17/22 patients, 77%).

Limitation of our study includes the small numbers of studied patients and we need further investigation to confirm the real utility in the diagnostic approach of hyponatremia.

## Conclusions

In conclusion, our data confirm that a calculated parameter such as FEUA is an excellent marker to discriminate at admission SIAD vs. sodium depleted patients treated with furosemide and/or potassium canrenoate by avoiding the need for diuretic withdrawal or fluid challenge test. So otherwise, thiazide withdrawal, beyond obtaining a serum Na normalization, is still mandatory for differential diagnosis of the hyponatraemia in TIH vs. SIAD patients.
